# Community of thermoacidophilic and arsenic resistant microorganisms isolated from a deep profile of mine heaps

**DOI:** 10.1186/s13568-015-0132-5

**Published:** 2015-08-19

**Authors:** S Casas-Flores, E Y Gómez-Rodríguez, J V García-Meza

**Affiliations:** División de Biología Molecular, IPCYT, Camino a la Presa San José 2055, Lomas 4a, 78216 San Luis Potosí, SLP México; Geomicrobiología, Instituto de Metalurgia, UASLP, Sierra Leona 550, Lomas 2a, 78210 San Luis Potosí, SLP México

**Keywords:** Mine heaps, Bacterial diversity, Extremophiles, Arsenic resistance system

## Abstract

Soluble arsenic (As) in acidic feed solution may inhibit the copper (Cu) bioleaching process within mine heaps. To clarify the effect of soluble arsenic on the live biomass and bioxidative activity in heaps, toxicological assays were performed using a synthetic feed solution given by a mine company. The microorganisms had previously been isolated from two heap samples at up to 66 m depth, and cultured using specific media for chemolithotrophic acidophiles (pH 1–2) and moderate thermophiles (48°C), for arsenic tolerance assay. The four media with the highest biomass were selected to assay As-resistance; one culture (Q63h) was chosen to assay biooxidative activity, using a heap sample that contained chalcopyrite and covellite. We found that 0.5 g/L of As does not affect living biomass or biooxidative activity on Cu sulfides, but it dissolves Cu, while As precipitates as arsenic acid (H_3_AsO_4_·½H_2_O). The arsenic tolerant community, as identified by 16S rDNA gene sequence analysis, was composed of three main metabolic groups: chemolithotrophs (*Leptospirillum*, *Sulfobacillus*); chemolithoheterotrophs and organoheterotrophs as *Acidovorax temperans*, *Pseudomonas alcaligenes*, *P. mendocina* and *Sphingomonas* spp. *Leptospirillum* spp. and *S.thermosulfidooxidans* were the dominant taxa in the Q63–66 cultures from the deepest sample of the oldest, highest-temperature heap. The results indicated arsenic resistance in the microbial community, therefore specific primers were used to amplify *ars* (arsenic resistance system), *aio* (arsenite oxidase), or *arr* (arsenate respiratory reduction) genes from total sample DNA. Presence of *arsB* genes in *S*. *thermosulfidooxidans* in the Q63–66 cultures permits H_3_AsO_4_-As(V) detoxification and strengthens the community’s response to As.

## Introduction

A mine heap is an imposing man-made “deposit” where crushed ore and low-grade minerals are agglomerated and stacked to a height of up to 100 m and hundreds of meters in length and width. Within such mine heaps, bioleaching processes occur. The commonly used process of bioleaching is feeding a heap with an acidic (pH < 2) “leach solution”, to promote chemolithotrophic activity of thermoacidophilic microorganisms living in the heap; the microorganisms then catalyze the solubilization or bioleaching of economically valuable metals, as Cu (Rawlings and Johnson 2007). Bioleaching in mine heaps is a widely-employed process used to extract metals from low-grade ores, which would not be economically extracted by any other method.

In a Cu mine in northwest Mexico, temperatures up to 85°C in the mine heap are generated because of the exothermic biooxidation of metal sulfides (MS) as chalcopyrite (CuFeS_2_). Because of the temperatures, thermophiles (moderate, living at 35–60°C; or extreme, 60–90°C) may be the dominant microorganisms in these mine heaps. Because of the huge spatial–temporal heterogeneity of mine heaps in terms of characteristics as mineral reactivity, irrigation efficiency, temperature, pH, and partial pressure of O_2_, CO_2_, redox potential, dissolved solutes, and available nutrients, considerable diversity of resident acidophilic Archaea and Bacteria is expected, including auto- or heterochemolithotrophs, aerobes, microaerophiles, or anaerobes in the deepest zones (Demergasso et al. 2005; Remonsellez et al. 2009).

However, the mining industry has been concerned about the presence of soluble As(III)/As(V) in feed solution, which may affect the chemolithotrophic microorganisms that inhabit the heaps, thereby affecting the overall Cu bioleaching process. Soluble As may be present because it is a “chalcophile element” with a low affinity for oxygen, and preferentially bonding to sulfur to form sulfides (Oremland and Stolz 2003). As-containing MS (e.g., arsenopyrite FeAsS, or enargite Cu_3_AsS_4_) are common in Cu-sulfide ore deposits. Thus, As-bearing phases are generated, and are present in solid and liquid waste from diverse mining processes, including roasting of the powdered enriched ore (producing fine particle waste), conversion of smelting products (dusts), the solvent extraction process (solutions), Cu recovery by refining (electrolytes), precious metals refinery (purges) and heap leaching [acid mine drainage (AMD)]. The concentration of arsenic in AMD, similar to that of mine heaps, may be from 2 to 20 g/L (Santini et al. 2000; Bednar et al. 2002; Delavat et al. 2012a, b).

Some microorganisms are able to use As compounds as electron donors or electron acceptors (Ehrlich 1996), including arsenite [As(III)] oxidizers and arsenate [As(V)] reducers, which have been described since 1918, but particularly during the last decade (Battaglia-Brunet et al. 2002, 2006; Dopson et al. 2003; Oremland and Stolz 2003; Baker-Austin et al. 2007; Muller et al. 2007; Bryan et al. 2009; Corsini et al. 2010; Delavat et al. 2012a; Travisany et al. 2012; *inter alia*). As(III) is more toxic than As(V). As(III) binds to the thiol groups of proteins, interfering with the function of several enzymes such as pyruvate dehydrogenase and 2-oxo glutarate dehydrogenase (Jackson and Dugas 2003; Oremland and Stolz 2003); As(III) also depletes intracellular glutathione (a tripeptide that acts as antioxidant and protects cells against free radicals), provoking the oxidation of the cytosol (Ehrlich 1996). As(V) acts as a structural analog of phosphate, and replaces the phosphate in cellular processes, inhibiting a plethora of biological phosphate-dependent reactions, including oxidative phosphorylation and production of ATP (Ehrlich 1996; Jackson and Dugas 2003). This explains greater tolerance to As(V) than to As(III): for example, strains of *Pseudomonas* and *Bacillus* may tolerate 13 mM As(V), but only 10 mM As(III) (Shakya et al. 2012); similarly, bacteria isolated from an arsenic-contaminated river in the Atacama Desert (Chile) are tolerant to As(V), from 100 to 1,000 mM; but only tolerate As(III) in the range of 2–40 mM (Escalante et al. 2009).

Conversely, it is well known that chemolithotrophs can oxidize MS under extreme conditions of pH (pH < 3), temperature (over 45°C) and metal-rich media, which may contain high concentrations of toxic elements such as As (Rawlings and Johnson 2007). In the acidic (pH 3.1), geothermal (58–62°C) springs of Yellowstone National Park (USA), soluble As(III) is oxidized predominantly by microbial mats (Jackson et al. 2001) to amorphous Fe(III)/As(V)-rich coprecipitate phases (Langner et al. 2001). At the Carnoulès mine in France, there has been rapid coprecipitation of large amounts of As with Fe(III) in bacterial mats, as tooeleite [Fe_6_(AsO_3_)_4_-(SO_4_)(OH)_4_·4H_2_O], mineral ferric arsenite sulfate oxyhydroxide, and amorphous mixed As(III)/As(V)-Fe(III) oxy-hydroxide compounds (Morin et al. 2003).

Arsenic resistance is an important capability for bioleaching microorganisms, because As is released from minerals such as arsenopyrite during bioleaching (Dopson et al. 2003). Over the last decade, many As-resistant microorganisms have been isolated. Specifically, diverse chemolithotrophic As-oxidizers couple the oxidation of As(III) to the reduction of either oxygen (aerobically) or nitrate (anaerobically) and use the energy gained to fix CO_2_ or bicarbonate (HCO_3_^−^) into organic compounds (Santini et al. 2000; Dopson et al. 2003; Oremland and Stolz 2003; Rhine et al. 2008). *Acidithiobacillus ferrooxidans* CC1 precipitated arsenic unexpectedly, as arsenite instead of arsenate (Duquesne et al. 2003).

To elucidate the effect of As on the biooxidative activity of previously isolated communities of chemolithotrophic microorganisms (living biomass) from samples of two mine heaps (northwest Mexico), toxicological and biooxidative assays were performed using a synthetic solution containing As. The isolated biooxidizer and As tolerant microorganisms were identified by 16S rDNA sequencing from enriched cultures of the original samples. Because our results indicated the presence of As resistance, putatively because of the presence of *ars* (the arsenic resistance system gene), *aio* (arsenite oxidase), or *arr* (arsenate respiratory reduction), we searched for the presence of *aio*, *arsB*, and *arr3* in organisms in the enriched cultures. ArsB and Arr3 are arsenite carrier efflux proteins (Achour et al. 2007; Lett et al. 2012).

## Materials and methods

### Sampling and enrichment of microbial cultures

At a copper (Cu) mine company in Mexico NW, two mine heaps were sampled by drilling a core from the top to 54 m (heap “T”) and 66 m (heap “Q”) depth; composites of ca. 1 kg were made with samples taken every 9 m. Fifteen samples were obtained in all. Samples were then transferred to sterile plastic bags, which were then sealed.

To make enrichment microbial cultures in order to evaluate As tolerance, two culture media were selected for chemolithoautotrophs and chemolithoheterotrophs. From each of the 15 samples, 5 g was transferred to a test tube containing minimal salt-enriched medium for chemolithoautotrophic acidophiles (living at pH 1–2) and moderate thermophiles (living at 48°C). Although the mineral samples from the heaps comprise mainly iron sulfurs (pyrite FeS_2_, and chalcopyrite CuFeS_2_) and sphalerite (ZnS), we decided to use such media to ensure the development of microorganisms. The minimal salt medium was prepared by adding 0.5 g/L MgSO_4_·7H_2_O, 0.4 g/L (NH_4_)_2_SO_4_, 0.25 g/L K_2_HPO_4_, and 0.1 g/L KCl to distilled water. Finally, 2 g/L or 10 mg/L of FeSO_4_·7H_2_O were added for autotrophic or heterotrophic growth, respectively. Sulfur (S) sources were omitted, because of the S in the heap samples. For heterotrophic growth, the minimal salt medium was supplemented with yeast extract (2% w/v). The pH was adjusted to 1.8 with concentrated H_2_SO_4_, and the medium was sterilized at 120°C for 20 min. In total, 60 cultures (15 samples × 2 cultures, in duplicate) were made.

Tubes were incubated in an orbital shaker at 48°C and 170 rpm (Lumistell^TM^). Samples were taken from test tubes once a week, over 3 months, and observed under a Leica DME microscope, with the amount of biomass recorded, evaluating motility (using light microscopy) and the response to 5-ciano-2,3-ditolyl tetrazolium chloride (CTC; using fluorescence microscopy). Culture medium (10–20 mL) was added every week to ensure the continued presence of nutrients. After 3 months, the four cultures that had the highest content of live cells were selected to assay the effect of As on biomass (As tolerance assays). These were the chemolithoautotrophic communities from T27 and T54 (from heap “T”, samples obtained at 27 and 54 m depth, respectively), and the chemolithoheterotrophic communities Q18 and Q63 (heap “Q”, at 18 and 63 m depth, respectively).

### As tolerance assays

Culturing experiments were performed to assess the As tolerance of the selected microbial communities. Triplicate cultures were incubated in test tubes, shaken at 120 rpm and 48°C, for 7 days. Microorganisms were inoculated into a concentrated, synthetic feed solution that had been obtained from the mining company. The synthetic solution is a mixture of acids solutions and arsenic trioxide (As_2_O_3_) that is added to the typical feed solution for the mine heaps.

For the biotic assays, the synthetic solution was diluted to 10% concentration with minimal salt-enriched medium for autotrophic or heterotrophic growth (recipe given above); final concentrations of As and Cu were: 0 (no addition: control assays), or 0.88 g/L As (11.7 mM As) and 3.2 g/L Cu (50 mM Cu) (experimental assays). The pH was adjusted to 1 with H_2_SO_4_. The dilute synthetic solution also contained 0.24 g/L Fe (in the experimental biotic assays only).

Microbial growth was arbitrarily defined as an increase in observed living (moving) cells, or living biomass. Living biomass was recorded on days 0 (before As and Cu addition), 4, and 7, based on a previously determined growth curve (that had been obtained from cultures growing without As). The final As and Cu concentrations (on day 7) were analyzed with flame atomic absorption spectrophotometers (Perkin-Elmer 2380 and Perkin-Elmer 1100B).

### Biooxidative activity assay

To assess the biooxidative activity of a microbial community in the presence of As, the chemolithoheterotrophic community Q63h was chosen because of its high biomass production relative to the other cultures. In sterile Erlenmeyer flasks, ca. 1.6 × 10^6^ cells/mL from community Q63h were inoculated into minimal growth medium with added yeast extract. A sample from the mine heap Q63 (7% w/v) was also added. The heap sample had previously been dried for 48 h under ultraviolet (UV) light, with the sample spread evenly over a crystal surface in a laminar flow cabinet. The dispersed heap sample was shaken every hour to homogenize the exposure of grains. Synthetic solution, containing As and Cu, was added to the culture medium after the start of the assay to a final concentration of 10%. The final concentrations of As, Cu, and Fe were 0.5, 1.69, and 0.14 g/L, respectively. The flasks were incubated at 48°C, rotating at 120 rpm, for 21 days. Uninoculated control flasks were also incubated, in triplicate.

Before being added to the culture flasks, the UV-dried heap sample had been analyzed for major mineralogical phases using X-ray diffraction with Cu-Kα radiation (XRD, Rigaku DMAX 2200; Rigaku program Version 1.3). Mineralogical phases of fine particles (<2 µm) were examined using scanning electron microscopy (SEM; Philips XL30) coupled to an energy-dispersive spectroscopy detector (EDS; EDAX DX-4 CDU-LEAP). The detected mineralogy of the heap sample is given in Table 1. The pH and soluble Cu and As of the culture medium were analyzed before and after days 7, 14, and 21 of the assay. Living biomass was examined using light microscopy on days 0 (before As addition), 7, 14, and 21.

**Table 1:** Mineralogy of heap sample Q63–66

DRX analysis: Main minerals (>3%)	Quartz, SiO_2_ Illite, (KH_3_0)Al_2_Si_3_AlO_10_(OH)_2_
SEM analysis: Minor minerals (<3%)	Chalcopyrite, CuFeS_2_ Covellite, CuS Fe and Mg oxides Silicates of Fe, Al, Mg, Na, and K

### PCR amplification of 16S rDNA and generation of clone libraries

Total genomic DNA extractions from the selected enrichment cultures used for As tolerance assay (T27, T54, Q18h, Q63h); DNA extraction were performed using the harsh lysis method described in Gabor et al. (2003), with minor modification: samples were centrifuged at 3,000 for 15 min to concentrate biomass, lysis buffer and zircon/silica beads were added to the pellet, and the samples were vortex-agitated for 10 min. Extracted genomic DNA was used as a template for PCR amplifications of the 16S rDNA in a total volume of 25 μL containing 1× PCR buffer, 200 μM dNTPs, 2.5 mM MgCl_2_, 1 μM each of forward and reverse oligonucleotide primers, and 1 U of Taq polymerase (Promega). Universal primers 533F and 1492R were used to amplify bacterial 16S rDNA (Bond et al. 2000). Expected amplicon size was approximately 900 bp. Negative controls without DNA were included. A Touch gene Gradient or TC-412 thermocycler (Techne) was used for PCR; with an initial denaturing step at 94°C for 5 min; followed by 35 cycles of 94°C for 1 min, 55°C for 1.5 min, and 72°C for 2 min; with a final extension step at 72°C for 10 min. All PCR products were checked in a 1% (w/v) agarose electrophoresis gel in 1× TAE buffer, post-stained with ethidium bromide, and photographed.

Amplicons were cloned according to the manufacturer’s protocol in the pGEM-T Easy kit (Promega) and Sanger-sequenced using an ABI sequencer (Applied Biosystems). Sequences were compared with those in the NCBI database using BLAST (Altschul et al. 1990). A total of 80 clones (20 per culture) were sequenced and identified.

### Amplification of genes involved in arsenic resistance

Examining the communities used for the As tolerance assays, four pairs of oligonucleotides were used (Table 2) to attempt to amplify *aio*, *arsB*, and/or *arr3*. Total sample DNA was used as the template in the PCR as described above, using the primer pairs described in Table 2. The PCR had an initial denaturing step at 94°C for 5 min; followed by 35 cycles of 94°C for 45 s, 50°C for 45 s, and 72°C for 50 s; with a final extension step at 72°C for 10 min. All PCR products were checked on a 1% (w/v) agarose electrophoresis gel in 1× TAE buffer, which was post-stained with ethidium bromide and photographed. Amplicons were cloned as previously described.

**Table 2:** PCR primers for genes that confer resistance to arsenic

Name	Sequence 5′–3′	Gene	References
Primer #1F	5′-GTSGGBTGYGGMTAYCABGYCTA-3′	*aioB* (formerly *aoxB*)	Inskeep et al. (2007)
Primer #1R	5′-TTGTASGCBGGNCGRTTRTGRAT-3′		
darsB1F	5′-GGTGTGGAACATCGTCTGGAAYGCNAC-3′	*arsB*	Achour et al. (2007)
darsB1R	5′-CAGGCCGTACACCACCAGRTACATNCC-3′		
dacr1F	5′-GCCATCGGCCTGATCGTNATGATGTAYCC-3′	*arr3* (formerly *ACR3* 1)	
dacr1R	5′-CGGCGATGGCCAGCTCYAAYTTYTT-3′		
dacr5F	5′-TGATCTGGGTCATGATCTTCCCVATGMTGVT-3′	*arr3* (formerly *ACR3* 2)	
dacr4R	5′-CGGCCACGGCCAGYTCRAARAARTT-3′		

*aio* arsenite oxidase, *ars* arsenic resistance system, *arr* arsenate respiratory-reduction.

### Phylogenetic analysis

Sequences were classified using the NCBI Basic Local Alignment Search Tool (BLAST; http://www.ncbi.nlm.nih.gov). Sequences were aligned with those obtained from GenBank, using Clustal W v1.82 (Higgins et al. 1994; http://www.ebi.ac.uk/clustalw). Phylogenetic analyses of 16S rDNA and *arsB* sequences were done in MEGA4 (Tamura et al. 2007) using neighbor joining (Saitou and Nei 1987); evolutionary distances were computed using the maximum composite likelihood method (Tamura et al. 2004), based on the number of base substitutions per site.

## Results

### As tolerance and biooxidative assays

To select the moderately thermophilic and acidophilic communities that grow best in specific culture media, we examined 3-month-old cultures microscopically. Bacilli were present in 30% of the analyzed cultures (18 flasks). Four of these cultures, with the highest biomass content, were selected (from heap samples T27, T54, Q18, and Q63; data not shown) to assay the effect of As on biomass.

**Fig. 1:**
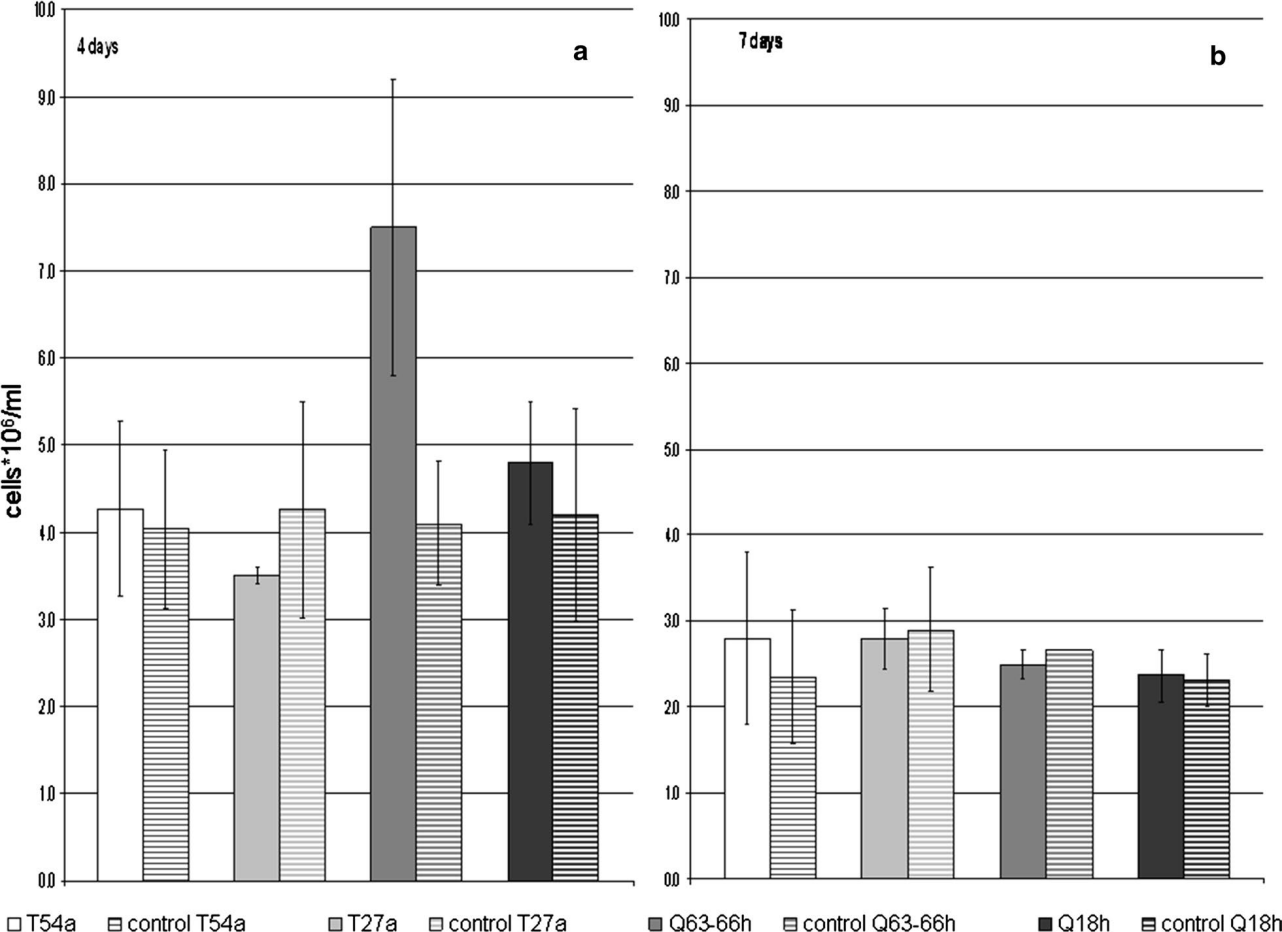
Microbial biomass after 4 (**a**) and 7 (**b**) days in cultures with samples obtained from two mine heaps (T and Q), at depths of 18 (Q18), 27 (T27), 54 (T54) and 63 (Q63) meters. *h* cultured with yeast extract, 2% w/v. *Solid columns* with As; *columns with horizontal lines* controls without As; *bars* standard errors.

According to the growth curves calculated for each selected culture, maximum biomass was reached at day 4 (Fig. 1). After 4 days, the biomass of the cultures containing 0.88 g/L As (11.7 mM) was not significantly different from that in the controls. The only exception was the Q63 culture, where biomass was significantly higher than in its corresponding control (Fig. 1a). At day 7, biomass decreased both in experimental (with As) and control (without As) cultures, with no significant difference between them (Fig. 1b). The main differences between experimental and control cultures were that in the experimental cultures, the pH decreased to <1, and As-rich precipitate was observed at the bottom of each tube on day 7.

**Fig. 2:**
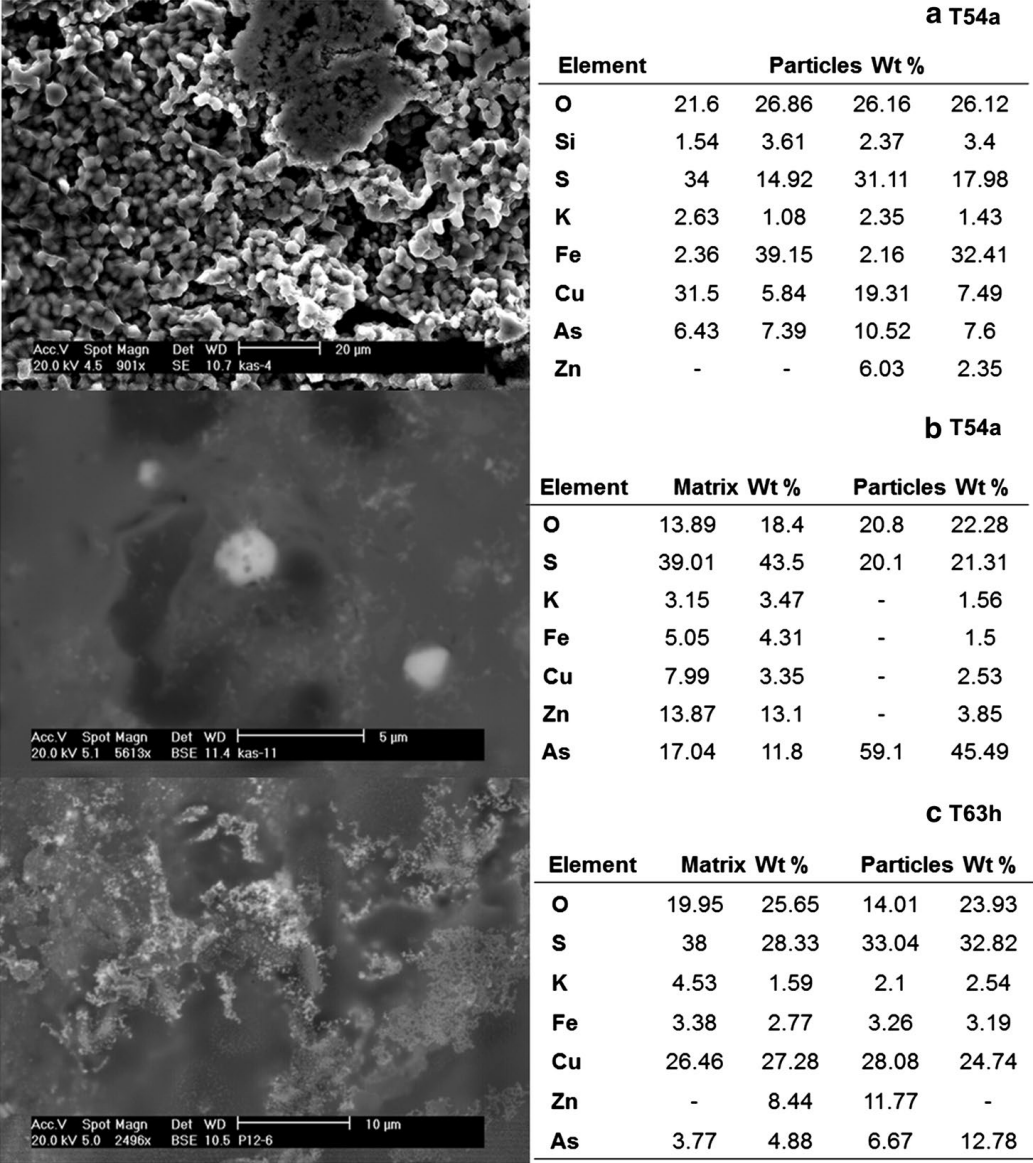
Scanning electron micrograph and SEM-EDAX analysis of the precipitates formed after 7 days of As tolerance assays, using media for autotrophic (**a**) and heterotrophic (**b**) growth. The data show the minimum and maximum As (%wt) obtained.

In the tubes prepared for autotrophic growth, the precipitates included copper sulfate, and iron (hydro)oxides wherein As may be adsorbed (Fig. 2a). These cultures also contained arsenic (Fig. 2b).The precipitates of heterotrophic trials contained more particles of copper but fewer of As (Fig. 2c) than in the autotrophic trials.

**Fig. 3:**
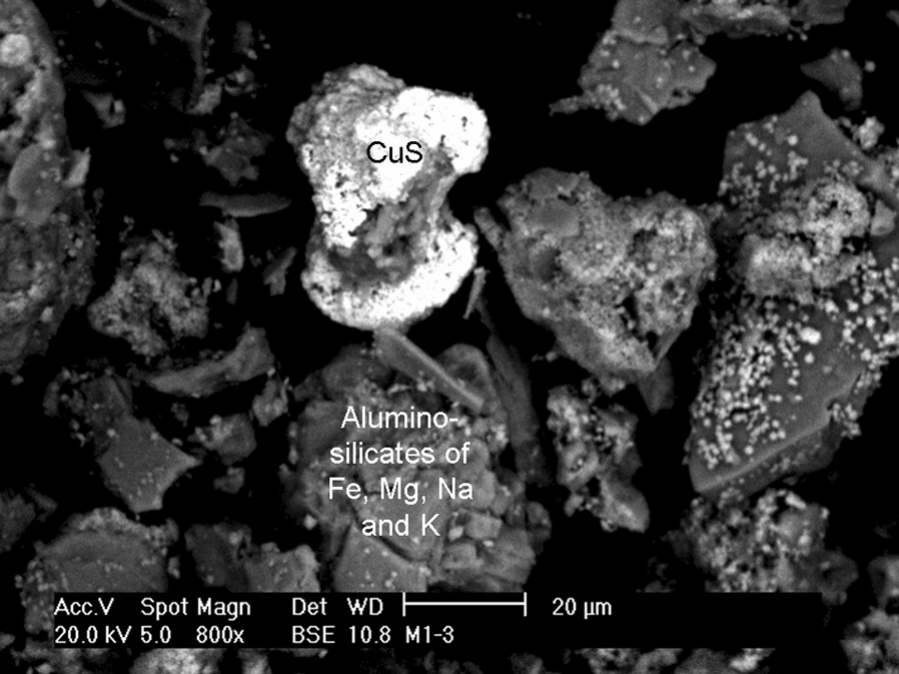
Scanning electron micrograph and SEM-EDAX analysis of the UV-dried mine heap sample added to Erlenmeyer flasks during the biooxidative assays.

For the biooxidative assay in the presence of 0.5 g/L As (6.7 mM, added to the culture medium), heap sample Q63 was chosen, because no arsenic minerals had been detected therein, but chalcopyrite (CuFeS_2_) and the secondary Cu mineral, covellite (CuS; Fig. 3; Table 1) had been detected. Notably, microorganisms (~10^4^ cells/mL) were observed in the control non-inoculated flasks after 21 days, because the heap sample was not entirely sterilized by UV.

**Fig. 4:**
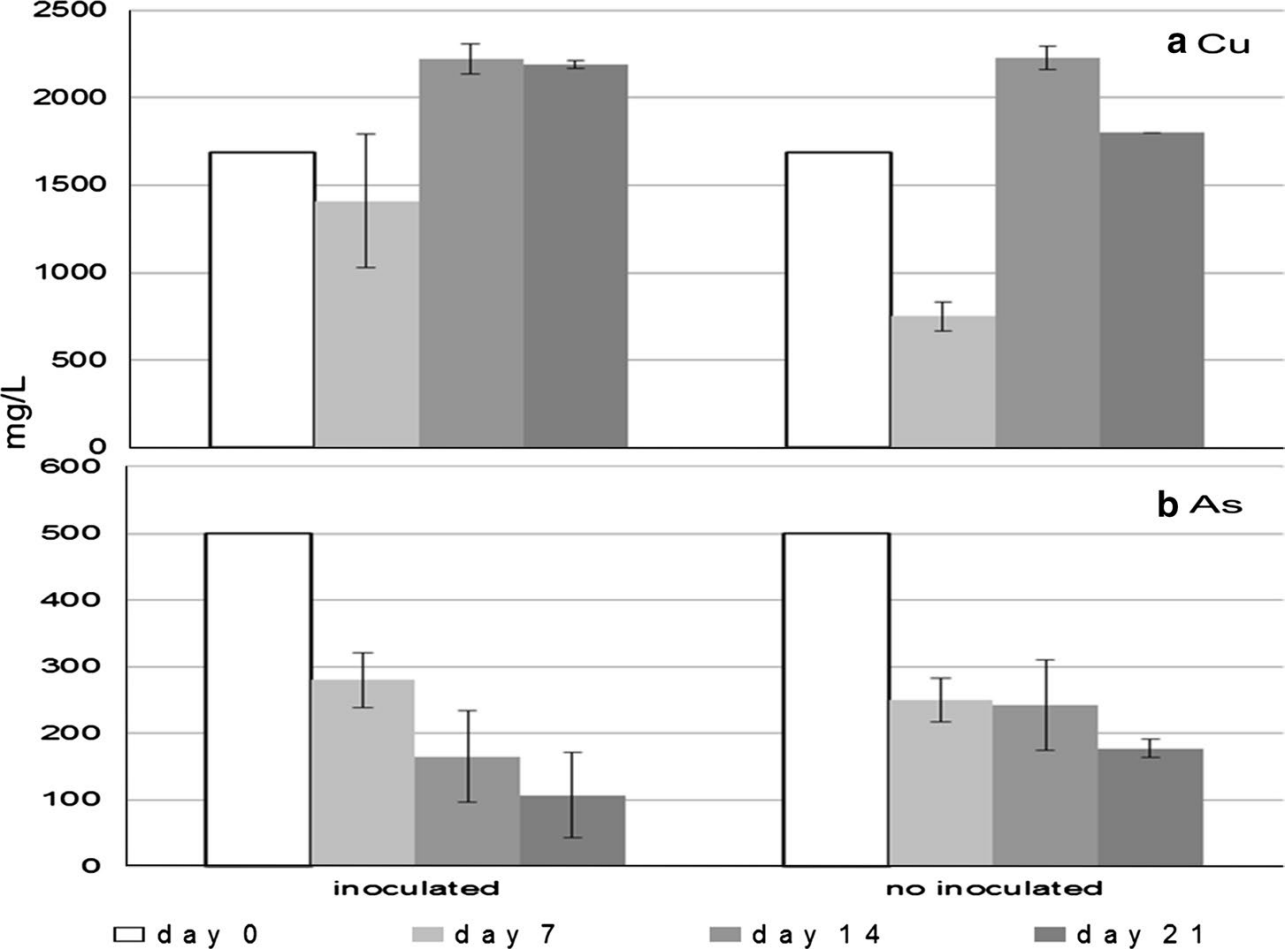
Soluble Cu (**a**) and As (**b**) after 0 (initial), 7, 14, and 21 days of (bio)oxidative assays, in inoculated (experimental) and non-inoculated (control) trials (*solid and dotted lines*, respectively).

Experimental cultures that had been inoculated with microorganisms from the chemolithoheterotrophic community Q63h, exposed to the heap sample in the presence of As for 21 days, had more Cu leached (64.2% Cu removed) from Cu sulfides than the non-inoculated controls (48.7% Cu removed) (Fig. 4a). Copper leaching is mediated by acidic dissolution of covellite. On day 21, the concentration of soluble As had decreased by 78.6% in experimental, inoculated flasks, and by 63.4% in control, non-inoculated flasks (Fig. 4b). These results show that the precipitation of As occurs independently of the biomass present. By day 21, the pH had increased slightly, from 1 up to 1.4, while the Eh decreased slightly, from 700 to 650 mV, because of the decrease in Fe(III) concentration. Under these conditions, As precipitates as arsenic acid (H_3_AsO_4_).

### Microbial community

To identify the microorganisms used in As tolerance assays, four 16S rDNA libraries were constructed and sequenced from samples T27, T54, Q18h, and Q63h. Table 3 summarizes the samples in which microorganisms were identified as well as the GenBank accession numbers of the BLAST matches.

**Table 3:** Classification based on BLAST results of bacterial 16S rRNA sequences present in enriched cultures

Microorganism	Comments	Heap (frequency)
*Acidobacteria* bacterium sp.^a^	Chemoorganotrophic; from acidic mineral environment; non-cultured soil bacterium present in radionuclide- and metal-contaminated environments	T27 (1), Q63–66h (5)
*Acidovorax temperans*	Type strain CB2; isolated from an activated sludge wastewater treatment plant in northern New Zealand; forms biofilms, promotes flocculation, removes phosphorous	T27 (2)
*Acidovorax* spp.^a^	Strain JS42; from soil; aerobic; capable of degrading toxic nitroaromatic compounds and polyethylene; As resistance	T54 (2)
*Bacillus* spp.	Widely distributed bacterium; highly resistant to As(III)	Q63–66h (1)
*Brevibacillus borstelensis*	From soil; thermophilic; lipolytic; degrades polyethylene as C source; in acidic soil waste; previously described in leaching ponds in Cananea Mine, Mexico	T54h (2)
*Leptospirillum ferriphilum* ^a^	AMD; bioleaching tank for polymetallic (Cu, Zn and Fe sulfides) concentrates; mine heaps and leaching (pregnant solution) ponds; previously described in leaching ponds of Cananea Mine, Mexico; As resistance	Q63–66 (1)
*Leptospirillum* spp.^a^	Bioleaching tanks, mine heaps and bioleaching (pregnant solution) ponds; Ni mine tailings; As resistance	T27 (2), T54 (2), Q63–66 (3)
*Paenibacillus* spp.	*P. polymyxa* has been studied for bioflotation and bioflocculation; as a soil inoculant in agriculture and horticulture; reported in rock varnish; fixes N; degrades cellulose; some species reduce nitrate; As resistance	T27 (1), T54 (2), Q63–66h (2)
*Pseudomonas alcaligenes*	Widely distributed; used as soil inoculant for bioremediation purposes; degrades polycyclic aromatic hydrocarbons	T27 (1), T54 (2), Q63 (2)
*P. mendocina* HQ113219.1	Anaerobic; solubilizes Fe minerals; from soil enrichment with ethanol; degrades acyclic isoprenoids; As resistance	T54 (2)
*Pseudomonas* spp.	Anaerobic; As resistance	Q18h, T54 (2)
*P.* *pseudoalcaligenes*	Widely distributed; anaerobic; from soil; uses cyanide as N source	T27 (1), T54 (1), Q63–66
*Sphingomonas* spp.	Chemoheterotrophic; strictly aerobic; some degrade chlorinated dibenzofurans and dibenzo-p-dioxins; reduces As(V) via *arsC*; Cu tolerant	Q18h (1), Q63-66h (2)
*Sulfobacillus acidophilus*	Mixotrophic; Fe^3+^ reducer; yeast extract as an electron source; geothermal and sulfur-rich environments	Q18h (1)
*Sulfobacillus* sp. 1^a^ and sp. 2	Mixotrophic for organic compounds (such as yeast extract); AMD; mine heaps; bioleaching tanks for polymetallic concentrates; As tolerant	Q18h, sp. 1 (5); Q63–66 (h), sp. 2 (1)
*S. thermosulfidooxidans*	Mixotrophic; geothermal environments; carbon deposits bioleaching tanks of pyrite, Co-pyrite, arsenopyrite, chalcopyrite and chalcocite; mine heaps and leaching ponds; As tolerant	Q18h (12), T27 (8), Q63–66h (13)

*h* cultured with yeast extract, 2% w/v.

^a^Non-cultivable species (NCBI).

The microorganisms that were identified belonged to Alphaproteobacteria (e.g., *Sphingomonas*), Betaproteobacteria (members of Comamonadaceae), Gammaproteobacteria (*Bacillus*, *Aeromonas*, *Pseudomonas*), Acidobacteria, Nitrospira (*Leptospirillum*), and Firmicutes (*Sulfobacillus*, *Paenibacillus*). BLAST searching returned significant identities with sequences of *Sulfobacillus* spp. (99%; GenBank Accession) and *Leptospirillum* spp. (83%), microorganisms that are directly responsible for bioleaching; other sequences with significant BLAST matches included *Paenibacillus* spp. (100%), *Pseudomonas* spp. (100%), *Acidobacteria* spp. (97%), *Aeromonas* spp. (100%), *Sphingomonas* spp. (100%), and a bacterium from Comamonadaceae (100%). Members of these taxa are commonly found in environments where leaching occurs (Remonsellez et al. 2009).

In the cultures incubated with yeast extract, the only species that grew were *Brevibacillus borstelensis* and *Sphingomonas* spp.; while *Pseudomonas alcaligenes*, *P*. *mendocina* and *Paenibacillus* spp. grew in cultures without yeast extract (Table 3).

### *arsB* gene

**Fig. 5:**
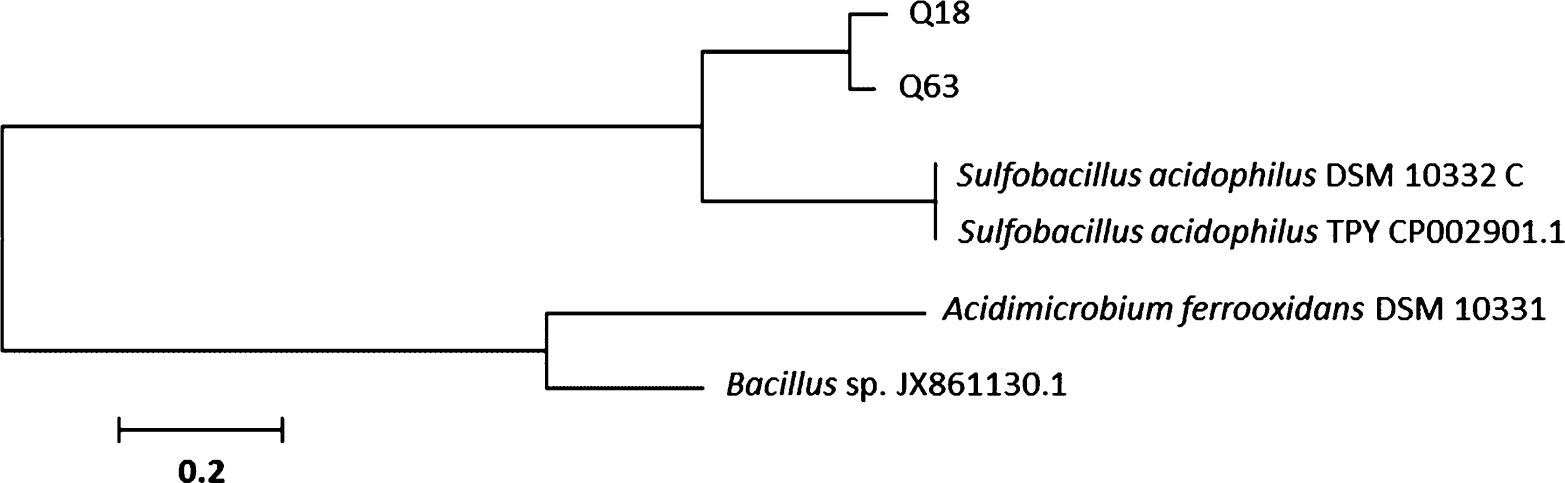
Maximum parsimony tree of *arsB* Permease sequences. The optimal tree with the sum of branch lengths = 2.73 is shown. The tree is drawn to scale, with branch lengths in the same units as those of the evolutionary distances used to infer the phylogenetic tree. The evolutionary distances are in the units of the number of base substitutions per site. All sequenced codon positions were included in the alignment, for introns and exons in *arsB*; however, every site containing gaps or missing data was then eliminated from the dataset. There were a total of 707 positions in the final dataset. *arsB* genes from different bacteria were used as indicated in the tree by their name, followed by the GenBank accession numbers. The consensus sequences of Q18 and Q63 samples were used to construct the tree.

**Fig. 6:**
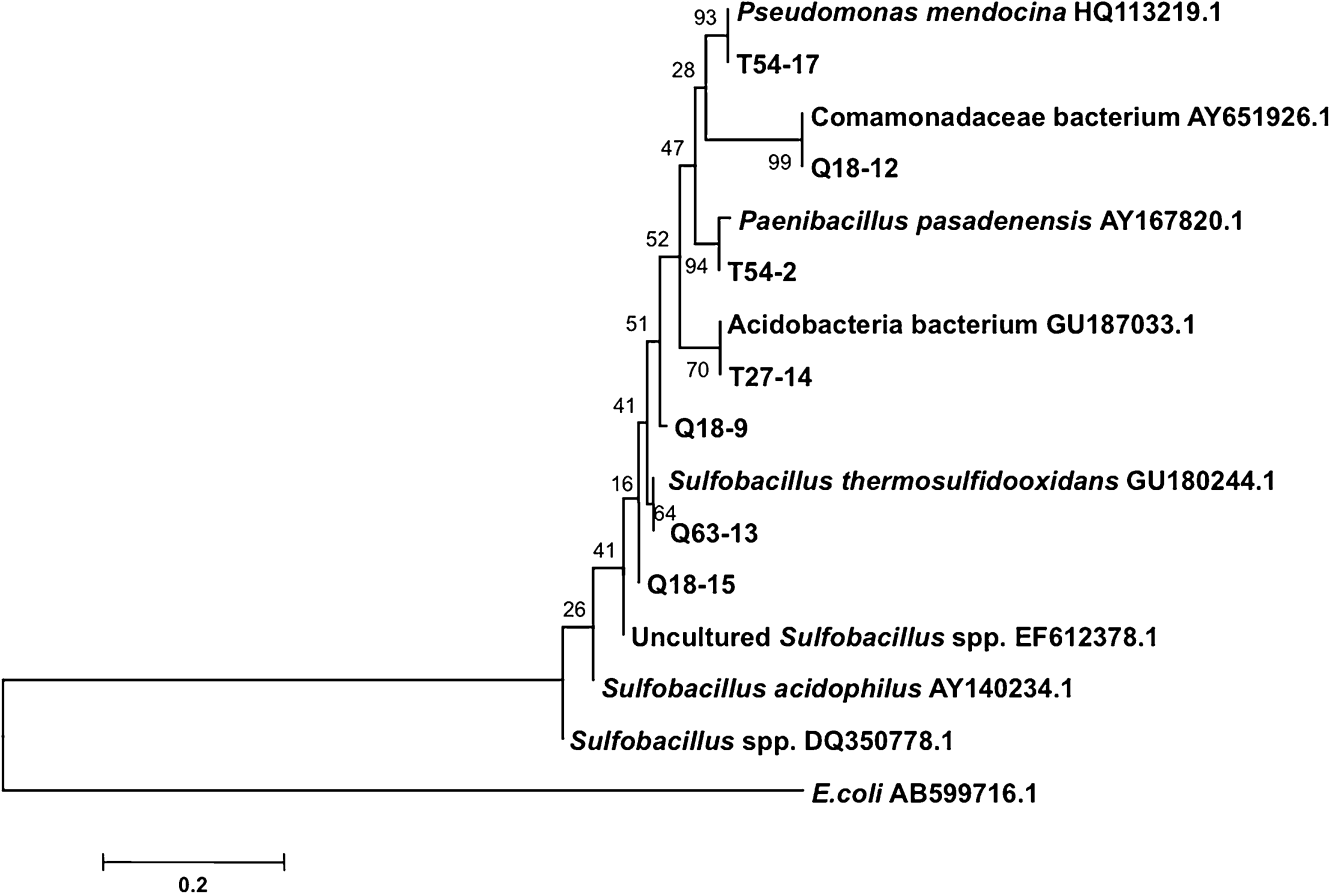
Neighbor-joining tree showing phylogenetic relationships among different microorganisms isolated from mine heaps, based on partial 16S rDNA sequences. *Node numbers* are support based on 500 bootstrap replicates. Representative 16S rDNA clones used for the analysis were: T54-17; Q18-12; T54-2; T27-14; Q18-9; Q63-13 and Q18-15. Sequences from NCBI GenBank used for the analysis were: *Pseudomonas mendocina* HQ113219.1; Comamonadaceae bacterium AY651926.1; *Paenibacillus pasadenensis* AY167820.1; *Acidobacteria* bacterium GU187033.1; *Sulfobacillus thermosulfidooxidans* GU180244.1; Uncultured *Sulfobacillus* EF612378.1; *Sulfobacillus acidophillus* AY140234.1; *Sulfobacillus* sp. DQ350778.1; *Escherichia coli* AB599716.1 was used as the out-group.

The *arsB* gene, an As efflux pump, was amplified from DNA samples from Q63h and Q18 communities, using primers darsBIF and darsBIR (Table 2). The sequences obtained were classified, using BLAST, as belonging to *Sulfobacillus* species, *S*. *thermosulfidooxidans* and *S*. *acidophilus*. Phylogenetic relationships of the *arsB* sequences (Fig. 5) are consistent with those in the 16S rDNA tree (Fig. 6).

## Discussion

The culturable communities of acidophiles and moderate thermophiles that inhabit two mine heaps (“T” and “Q”) of a Cu mine (Mexico NW) are resistant to As, and the presence of soluble As did not affect the ongoing bioleaching of Cu from chalcopyrite and covellite, as shown by the results of the biooxidative assays.

The biooxidative assays were performed using the culture derived from Q63–66, the deepest, highest-temperature sample, because this contained the most living biomass in the As-tolerance assays (Fig. 1). In this culture, the chemolithotrophs *Leptospirillum* spp. and *S.thermosulfidooxidans* were the dominant species. Both species are As-resistant (Watling et al. 2008; Dopson and Holmes 2014), and *arsB* has previously been identified in *Sulfobacillus* (van der Merwe et al. 2010). *arsB* drives efflux of As(III) through the cell membrane, via membrane potential (Gihring et al. 2003; Dopson et al. 2003; Slyemi and Bonnefoy 2012).

We propose that the presence of *arsB* genes from *S*. *thermosulfidooxidans* in the Q63–66 community (Fig. 5) strengthens the response to As. The cultures used for As tolerance assays contained arsenic acid (e.g., H_3_AsO_4_; Fig. 2b) formed from arsenic oxide, because of the prevalent oxidizing (Eh up to 1,000 mV/SHE) and acidic (pH 0.9–1.2) conditions during the assays (Ferguson and Gavis 1972). Thus, the response to As is likely via H_3_AsO_4_-As(V) detoxification, using the reduction of As(V) to soluble As(III) through the oxidation of dissolved HS^−^, both of which are processes mediated by microorganisms (Andrade et al. 2010; Eq. 1).

1 $$4 {\text{HAsO}}_{4}^{2 - } + {\text{HS}}^{ - } + 7 {\text{H}}^{ + } \to 4 {\text{H}}_{ 3} {\text{AsO}}_{3}^{0} + {\text{SO}}_{4}^{2 - }$$

Because of the prevalent oxidizing (Eh > 640 mV) and acidic (pH < 1.0) conditions in both assays (As tolerance and biooxidative), As(V) is the dominant arsenic species that would form mixed and amorphous As(V)-Fe(III) oxy-hydroxides, provided enough Fe(II) oxidizes (Morin et al. 2003). In cells, As(V) detoxification involves its enzymatic reduction to As(III) in the cytoplasm by arsenate reductase arsC; As(III) is then pumped out of the cell by ArsB (Slyemi and Bonnefoy 2012), and may precipitate into the medium via reactions catalyzed by microorganisms (Leblanc et al. 1996; Bednar et al. 2002; Casiot et al. 2003; Morin et al. 2003); arsenic acid was detected after the As-tolerance assays only in inoculated tubes (Fig. 2). As(III) may also be sequestered outside the cell in its extracellular polymers, in which the Fe(II) oxidation site also serves as the As(III) nucleation site (Muller et al. 2007; Slyemi and Bonnefoy 2012).

Notably, at the end of the As-tolerance experiment, the precipitates in tubes for heterotrophic growth contained less arsenic (Fig. 2c) than those in the tubes for autotrophic growth (Fig. 2b). This is likely because of reductive dissolution of iron (hydr)oxides by labile organic matter (Andrade et al. 2010), e.g., the yeast extract added to the heterotrophic tubes; or because of the predominance of *Leptospirillum* spp. in autotrophic cultures and the presence of *Sulfobacillus* spp. only in heterotrophic cultures.

The ferrooxidizers *Leptospirillum ferriphilum* and *Leptospirillum* spp. (Table 3) are strictly chemolithoautotrophic, and they were detected primarily in cultures for autotrophic growth and in samples from depths greater than 27 m. *Leptospirillum* is a common genus present in bioleaching tanks, mine heaps and bioleaching (pregnant liquor solution) ponds (Tuffin et al. 2006; Remonsellez et al. 2009) where its role in the community is crucial as *Leptospirillum* fixes CO_2_ and oxidizes NH_4_^+^, providing C and N sources to the rest of the microbial community. The “carbon fixation rates in ~100 mm thick biofilms from AMD are broadly equivalent to those achieved across the ocean photic zone” (Denef et al. 2010). *Leptospirillum* also generates energy and Fe(III) through Fe(II) oxidation, triggering an Eh decrease that drives arsenic precipitation at pH ~1.0.

Meanwhile, *Sulfobacillus* was the most common genus detected in cultures obtained from heap samples (34 clones), independent of their depth of the samples within the heap (18, 27, 54, or 63 m). *Sulfobacillus* was the unique genus recovered from cultures Q18h, and was the dominant genus recovered from cultures Q63h (h: with yeast extract). The only *arsB* sequences present in the sample are those of *Sulfobacillus* (Fig. 5).

Recently, Acosta et al. (2014) extracted RNA from the pregnant liquor solution samples, also found that *Leptospirillum* and *S. thermosulfidooxidans* are the most abundant thermoacidophiles in the deeper areas of heaps (up to 54 m); their relative abundance increases with the age of the heap, and where there is pH lower than 2, high Eh, and up to 3 g/L of Cu (Demergasso et al. 2005; Remonsellez et al. 2009). Similar conditions were present in our experiments of As tolerance and biooxidation, while temperature was 48°C

Although the identified microorganisms from cultures comprise nine genera of bacteria, only two genera, *Leptospirillum* and *Sulfobacillus*, are chemolithotrophs typically found un mine heaps or bioleaching tanks. The diversity of microbes detected here in mine heaps includes other metabolic functional groups of bacteria, some of them previously described in mining environments and geothermal areas (Table 3), including sequences from *Pseudomonas* spp. (8 clones), *Acidobacteria* spp. (6 clones), and *Paenibacillus* spp. (5 clones). Rawlings and Johnson (2007) have found that despite the significant (low) diversity of microorganisms inhabiting mine heaps, relatively few taxa play a major role in biomining processes. These bacteria may indirectly affect the overall MS bioleaching process via biological interactions (symbiosis) with the MS biooxidizers, as has been suggested by Watling et al. (2008); e.g., the firmicute *Paenibacillus* could potentially use sulfur and iron compounds as an energy source (Bond et al. 2000). *Paenibacillus* Q8 has polymer-degrading enzymes that confer it cellulolytic, hemicellulolytic and amylolytic activities under a broad pH range (4–10) and under high As(III) and As(V) concentrations (up to 750 and 1,000 mg/L, respectively) (Delavat et al. 2012b).

Interestingly, most of the microorganisms identified in our work can degrade different complex organic compounds (Leys et al. 2004; Hadad et al. 2005; Delavat et al. 2012b). These include *Acidovorax* spp. (degrading toxic nitroaromatic compounds), *Brevibacillus borstelensis* (polyethylene), *Pseudomonas alcaligenes* (polycyclic aromatic hydrocarbons), *P*. *mendocina* (acyclic isoprenoids), *Paenibacillus* spp. (sugar monomers and some polymers including hemicellulose, cellulose, and starch), and *Sphingomonas* spp. (chlorinated dibenzofurans and dibenzo-*p*-dioxins) (Table 3). The presence of such degradative metabolic capabilities in the organisms cultured from our samples may partially be explained by the disposal of organic solvents, such as kerosene-based solvents (from the “solvent extraction process”) into the heaps.

The main metabolic groups of Cu- and As-tolerant microorganisms detected in our cultures communities from mine heap samples are: (1) chemolithoautotrophs, employing oxidation of Fe^2+^ or reduced S-compounds (*Leptospirillum* or *Sulfobacillus*, respectively); (2) chemolithoheterotrophs, employing reduction of Fe^3+^ in microaerophilic conditions (*Sulfobacillus*); and (3) aerobic or anaerobic organoheterotrophs (*Acidovorax*, *Brevibacillus borstelensis*, *Pseudomonas alcaligenes*, *P*. *mendocina*, *Paenibacillus* and *Sphingomonas*). The same metabolic groups have previously been described by Baker and Banfield (2003) AMD, and it was suggested that a subset of the detected microorganisms could fix the nitrogen required by the community. *Leptospirillum ferriphilum* and *Paenibacillus* fix nitrogen (Table 3; Xie et al. 2011). The metabolic capabilities of the communities cultured from mine heaps (carbon and nitrogen fixation, aerobic and anaerobic carbon degradation, sulfur and iron oxidation, microaerophilic iron reduction, arsenic and Cu resistance) increase the community’s functional robustness. Xie et al. (2011) also reported similar common metabolic capabilities in five different AMDs from Cu mines. The coexistence of microorganisms with different environmental requirements (such as oxygen) in mine heaps is because some occur as biofilms on the surface (García-Meza et al. 2010).

Delavat et al. (2012b) suggested that “AMDs could be considered as reservoir of genes with potential biotechnological properties”. We share this conclusion for microorganisms resident in mine heaps as described here, with their various metabolic capabilities (Table 3). These could be used to implement solutions to environmental pollution problems associated with mining or other activities. Examples could include phosphorus removal by *Acidovorax temperans*; polyethylene degradation by *Acidovorax* spp. and *Brevibacillus borstelensis*; cyanide degradation by *Pseudomonas pseudoalcaligenes*, or chlorinated dibenzofuran and dibenzo-*p*-dioxin degradation by *Sphingomonas* spp.

In this work, *Leptospirillum* and *Sulfobacillus* were the only chemolithotrophs identified using the prepared cultures. Culturing was necessary for the assays performed; but it is indispensable to sequence directly from samples, as well as to combine geochemical, biochemical and microbiological techniques, especially the GeoChips and omics approaches, to gain more comprehensive knowledge of the active microbial functions within this manmade ecosystem and their response to As.

To elucidate the effect of As on the biooxidative activity of previously isolated communities of chemolithotrophic microorganisms (living biomass) from samples of two mine heaps (northwest Mexico), toxicological and biooxidative assays were performed using a synthetic solution containing As. The isolated biooxidizer and As tolerant microorganisms were identified by 16S rDNA sequencing from enriched cultures of the original samples. Because our results indicated the presence of As resistance, putatively because of the presence of *ars* (the arsenic resistance system gene), *aio* (arsenite oxidase), or *arr* (arsenate respiratory reduction), we searched for the presence of *aio*, *arsB*, and *arr3* in organisms in the enriched cultures. ArsB and Arr3 are arsenite carrier efflux proteins (Achour et al. 2007; Lett et al. 2012).

This paper describes for the first time microorganisms isolated from mine heaps samples of 63 m depth. The culturable communities includes relatively few taxa playing a major role in MS biooxidation, as *Sulfobacillus* spp. and *Leptospirillum* spp. The other bacteria may indirectly affect the overall MS bioleaching process via biological interactions with the MS biooxidizers. The isolated are As tolerant microorganisms, but the only *arsB* sequences present in the sample are those of *Sulfobacillus*, while *Leptospirillum* oxidize Fe(II) triggering an Eh decrease that drives arsenic precipitation at pH ~1.0. According to the literature review, most of the cultivable and identified microorganisms are reservoir of genes with potential biotechnological properties.
